# Age- and Sex-Specific Profiles of Self-Reported Functional Limitations, Exercise Behavior, and Objective Functional Fitness in Korean Older Adults: Evidence from Two Independent 2023 Data Sources

**DOI:** 10.3390/healthcare14213699

**Published:** 2026-11-01

**Authors:** Alchan Kim

**Affiliations:** Department of Sports & Technology, College of Convergence, Seokyeong University, 124 Seogyeong-ro, Seongbuk-gu, Seoul 02713, Republic of Korea; younal@skuniv.ac.kr; Tel.: +82-2-940-7210; Fax: +82-2-940-7616

**Keywords:** older adults, exercise behavior, physical fitness, functional limitations, functional capacity, mobility, health promotion

## Abstract

Background/Objectives: Self-reported function, exercise behavior, and performance-based fitness describe related but distinct aspects of health in later life. This study compared their age- and sex-specific descriptive profiles using independent Korean datasets. Methods: The 2023 National Survey of Older Koreans contributed 9951 self-respondents; the 2023 National Fitness Award (NFA) dataset contributed 27,440 assessment records, which may not represent 27,440 unique individuals. Age-by-sex profiles were analyzed separately; no individual-level linkage or direct between-dataset statistical comparison was performed. Results: Stair-climbing and 300 m walking limitations, and related objective performance indicators, generally showed less favorable values in older age groups. Exercise non-participation followed a less consistent pattern. Among men, the weighted prevalence was 47.16% (95% CI 43.29–51.04%) at ages 65–69 and 40.88% (36.64–45.13%) at ages 75–79; their exploratory age-group contrast did not remain significant after Holm adjustment (adjusted *p* = 0.066). Among women, non-participation was 42.47–45.67% across ages 65–79. Conclusions: The observed directional similarities are descriptive and cannot establish agreement, predictive validity, or the effects of exercise at the individual level. Exercise participation reports, task-specific difficulty questions, and objective fitness tests should be regarded as distinct information sources requiring evaluation within the same individuals.

## 1. Introduction

Physical function in later life is closely related to the ability to maintain independent daily living. Functional status can be assessed through self-report, which captures difficulties experienced in everyday settings, and through performance-based testing under standardized conditions. These approaches are related but not interchangeable. Self-report reflects a person’s perceived difficulty in interacting with their usual environment, whereas performance tests quantify the ability to complete a defined task. Studies assessing both approaches in the same individuals have reported discordance between self-reported and performance-based functioning, which may persist over time [1,2,3].

The Resistance and Ambulation components of the FRAIL scale query difficulty with stair climbing and walking, respectively [4]. The Korean FRAIL scale has demonstrated clinical feasibility and validity in Korean older adults [5]. A meta-analysis of frailty and physical fitness identified walking performance, lower body strength, and grip strength as relevant correlates of function [6]. Self-reported limitations in stair climbing and ambulation are therefore conceptually related to objective lower extremity and mobility-related performance.

Exercise behavior and physical fitness also represent different constructs. Exercise is planned and structured physical activity undertaken to maintain or improve fitness, whereas physical fitness refers to measurable physical performance attributes [7]. Reporting participation in exercise is therefore not equivalent to having high muscular strength or functional performance. In older adults, exercise type, intensity, frequency, cumulative exposure, chronic disease, and physiological reserve can differ substantially; as such, exercise participation and objective fitness will not necessarily display parallel age-specific profiles.

The 2023 National Survey of Older Koreans (NSOK) targeted adults aged 65 years or older living in ordinary residential settings across all 17 administrative provinces and metropolitan cities of the Republic of Korea, using a stratified cluster-sampling design with survey weights [8]. It included questions about difficulty climbing 10 stairs without assistance, difficulty walking 300 m without assistance, and usual exercise participation and frequency. In contrast, the 2023 National Fitness Award (NFA) Physical Fitness Measurement Statistics provide large-scale objective measurements obtained at National Fitness Award centers, including grip strength, the 30 s Chair Stand, and the 3 m target return test [9]. Previous studies using NFA data have described functional fitness characteristics, developed fitness prediction equations, and examined associations between grip strength and other fitness components in Korean older adults [10,11,12,13]. However, NFA records were derived from voluntary program participation and should not be interpreted as a probability sample representative of all older adults in Korea.

The two datasets could not be linked at the individual level. Accordingly, the analysis was restricted to independently estimated age-by-sex profiles rather than individual-level correlations, measurement agreement, prediction, or direct statistical comparisons between datasets. Previous studies have examined self-reported and performance-based functioning within the same individuals [1,2,3], while research using NFA data has primarily described functional fitness or relationships among performance measures [10,11,12,13]. Aligning the two 2023 data sources by age group and sex permits a descriptive examination of these different information domains, although their distinct sampling mechanisms remain an important constraint on interpretation.

The present study aimed to characterize age- and sex-specific profiles of self-reported functional limitations, exercise behavior, and objective functional fitness in Korean older adults. The analysis was guided by the expectation that stair-climbing difficulty and 30 s Chair Stand performance would show broadly consistent age-group directions because both involve lower extremity function. Difficulty walking 300 m and the 3 m target return test were also examined within the broader mobility domain, without assuming that they measure equivalent abilities. In contrast, exercise participation and frequency were expected to provide potentially different age-group patterns because exercise behavior and current physical performance are distinct constructs. These expectations guided the descriptive comparisons rather than formal tests of measurement agreement, pairing specificity, or interchangeability.

## 2. Materials and Methods

### 2.1. Study Design and Data Sources

This cross-sectional secondary analysis used de-identified data from the 2023 NSOK and the 2023 NFA Physical Fitness Measurement Statistics. Because individuals could not be identified or linked across datasets, the datasets were analyzed independently rather than merged. Reporting was guided by the Strengthening the Reporting of Observational Studies in Epidemiology (STROBE) statement [14].

The 2023 NSOK targeted adults aged 65 years or older living in ordinary residential settings across all 17 administrative regions of the Republic of Korea. The sample was selected using a stratified cluster design based on Population and Housing Census enumeration districts. A total of 977 sampling districts were surveyed, and weights were provided for population-level estimation [8]. Of 10,078 respondents in the public-use file, 9951 were self-respondents, and 127 were proxy respondents. Because the primary constructs in this study were self-reported functional limitations and exercise behavior, the 9951 self-respondents constituted the primary analytic sample. Sensitivity analyses included all 10,078 respondents.

The NFA dataset contained 27,440 assessment records for adults aged 65 years or older in 2023. The National Fitness Award program provides fitness assessments through designated centers, with 37 fitness certification centers reported nationwide for the 2023 reference year [9]. Participation was voluntary. The available analytic file did not contain center or regional identifiers, preventing an examination of the geographic distribution of the assessment records. It also lacked individual identifiers, so repeated assessments of the same person within the year could not be identified. Whether all individuals were community-dwelling could not be verified from the available data. Accordingly, the 27,440 observations were analyzed as assessment records rather than necessarily unique individuals. Because these records were not obtained through probability sampling, they were used to describe the assessed population and not to estimate physical fitness for the Korean older adult population as a whole.

### 2.2. Age and Sex

Age groups were harmonized as 65–69, 70–74, 75–79, and ≥80 years. NSOK included exact age, whereas the NFA analytic extract provided only these categories; exact age within the ≥80 year group was unavailable for NFA records. Both files encoded sex as two recorded categories, male and female. A common coding was applied to those categories without assuming that their underlying ascertainment establishes biological sex or gender identity in either dataset.

### 2.3. Self-Reported Functional Limitations

Self-reported functional limitations were defined using the two function-oriented domains of the FRAIL scale: Resistance and Ambulation [4,5]. Resistance was coded as present when a respondent reported difficulty climbing 10 stairs continuously without assistance. Ambulation difficulty was coded as present when a respondent reported difficulty walking 300 m without assistance. These items were not used to calculate a K-FRAIL total score or diagnose frailty; rather, they were analyzed independently as task-specific self-reported limitations related to lower extremity function and mobility [5,8].

### 2.4. Exercise Behavior

Exercise behavior was measured using the 2023 NSOK questions on usual exercise outside work-related activity [8]. The participation item counted exercise performed at least once a week for at least 10 continuous minutes per occasion. Exercise non-participation was the primary behavioral outcome. Only respondents reporting exercise answered the subsequent questions on exercise days per week and usual minutes per session (B12-1 and B12-2). Weekly exercise days were summarized among exercisers; an exploratory exercise volume descriptor was calculated as reported weekly days multiplied by reported minutes per session. It reflects self-reported time, not intensity or a validated dose of training. Respondents reporting no exercise were not assigned zero exercise days or minutes for these conditional analyses.

### 2.5. Objective Functional Fitness

The NFA analytic extract contained absolute grip strength (kg), 30 s Chair Stand repetitions, and the time to rise from a chair, travel around a target 3 m away, return, and sit down (3 m target return test). The Chair Stand is an established lower body performance measure [15]. The 3 m movement sequence resembles that of the Timed Up and Go test [16]; however, identical administration protocols could not be verified from the analytic extract. Higher grip strength and Chair Stand repetitions, and shorter 3 m times, indicate better performance. The 3 m test was treated only as an auxiliary measure of short-distance mobility, dynamic balance, and agility [9,11], not as a measure of sustained 300 m walking capacity. A 2 min step variable was not included in the supplied 2023 older adult NFA analytic file or its variable specification. Although a 6 min walk variable was present, only 183 of 27,440 records had valid measurements (0.67%), precluding useful age-by-sex analysis. Valid record counts were 27,438 for grip, 26,762 for Chair Stand, and 26,761 for the 3 m test (Supplementary Table S4). Source values recorded as ‘-’ were treated as missing, and no values were imputed.

### 2.6. Prespecified Functional Domains and Directional Descriptive Comparison

Before comparing the descriptive profiles, two conceptually related—but not equivalent—task pairings were specified: stair-climbing limitation with 30 s Chair Stand performance (lower extremity function) and 300 m walking limitation with the 3 m target return test (the broader mobility domain). The comparisons examined only whether age-by-sex group profiles showed similar directions: more reported difficulty alongside less favorable performance in older groups. Given that many physical indicators vary with age, such directional similarity was treated as descriptive context rather than evidence of pairing specificity or validation. Exercise behavior was inspected separately for different directions across age strata. No mismatched pair superiority, individual agreement, correlation, cross-dataset gradient difference, or causal effect was tested.

### 2.7. Statistical Analysis

Analyses used Python 3.13.5 and statsmodels 0.14.6. For the NSOK, provided standardized weights (s_wgt1) were applied to age-by-sex weighted prevalences of stair-climbing limitation, 300 m walking limitation, and exercise non-participation. Weighted binomial logistic models included age group, sex, and their interaction; sandwich covariance was clustered on the public file survey-district identifier (surveyNoR, 977 distinct districts). The overall interaction was assessed with a 3-df Wald test. To distinguish age-by-sex interaction from age-group variation within each sex, separate 3-df omnibus age-group Wald tests were performed for men and women for all three NSOK outcomes (Supplementary Table S6). For exercise non-participation, each older age group was additionally compared with the 65–69-year reference group within each sex. Odds ratios, unadjusted 95% confidence intervals, and both unadjusted and Holm-adjusted *p* values for these three comparisons within each sex are reported in Supplementary Table S3. Reported exercise days and weekly exercise minutes were summarized as survey-weighted means among exercisers only. The published NSOK documentation describes the original stratified clustered sample [8], but a dedicated stratum identifier was absent from the public analytic extract. Accordingly, weights and district clustering were represented, but not the full original design; resulting standard errors may differ from complete design-based estimates.

For NFA outcomes, age-by-sex means, standard deviations, and outcome-specific valid record counts were calculated. General linear models included age group, recorded sex, and their interaction, using HC3 heteroskedasticity-consistent covariance. Main effect Wald tests used sum-to-zero effect coding, and interaction effect sizes were summarized descriptively with partial eta squared (η^2^). Analyses used available records without imputation. A record identifier for repeated visits and center-level grouping was unavailable, limiting the inferential interpretation of robust standard errors. Tests were two-sided; analyses were exploratory and, other than the specified Holm-adjusted within-sex contrasts, *p* values were not adjusted for multiplicity. No statistical effect size, slope difference, or association was calculated across the two datasets.

## 3. Results

### 3.1. Analytic Samples

The primary NSOK analysis included 9951 self-respondents (3824 men; 6127 women), and the NFA file comprised 27,440 assessment records (14,081 male-coded; 13,359 female-coded). The source composition differed: men accounted for 44.04% of the survey-weighted NSOK sample versus 51.32% of NFA records. Within men, persons or records aged ≥80 years represented 19.17% of the weighted NSOK distribution and 10.01% of NFA records; the corresponding female values were 27.98% and 12.92% (Table 1; Supplementary Table S5). Because NFA entries are assessment records, these descriptive differences are not an estimate of fitness-related selection bias.

### 3.2. Age- and Sex-Specific Profiles of Self-Reported Functional Limitations

The weighted prevalence of stair-climbing limitation across ages 65–69, 70–74, 75–79, and ≥80 years was 10.79%, 17.21%, 26.05%, and 39.12% among men, and 14.97%, 26.15%, 40.89%, and 57.82% among women. Although these descriptive values were higher in older age groups of both sexes, evidence for a different age pattern by sex was insufficient (age-by-sex Wald χ^2^ = 6.20, df = 3, *p* = 0.102).

**Figure 1:**
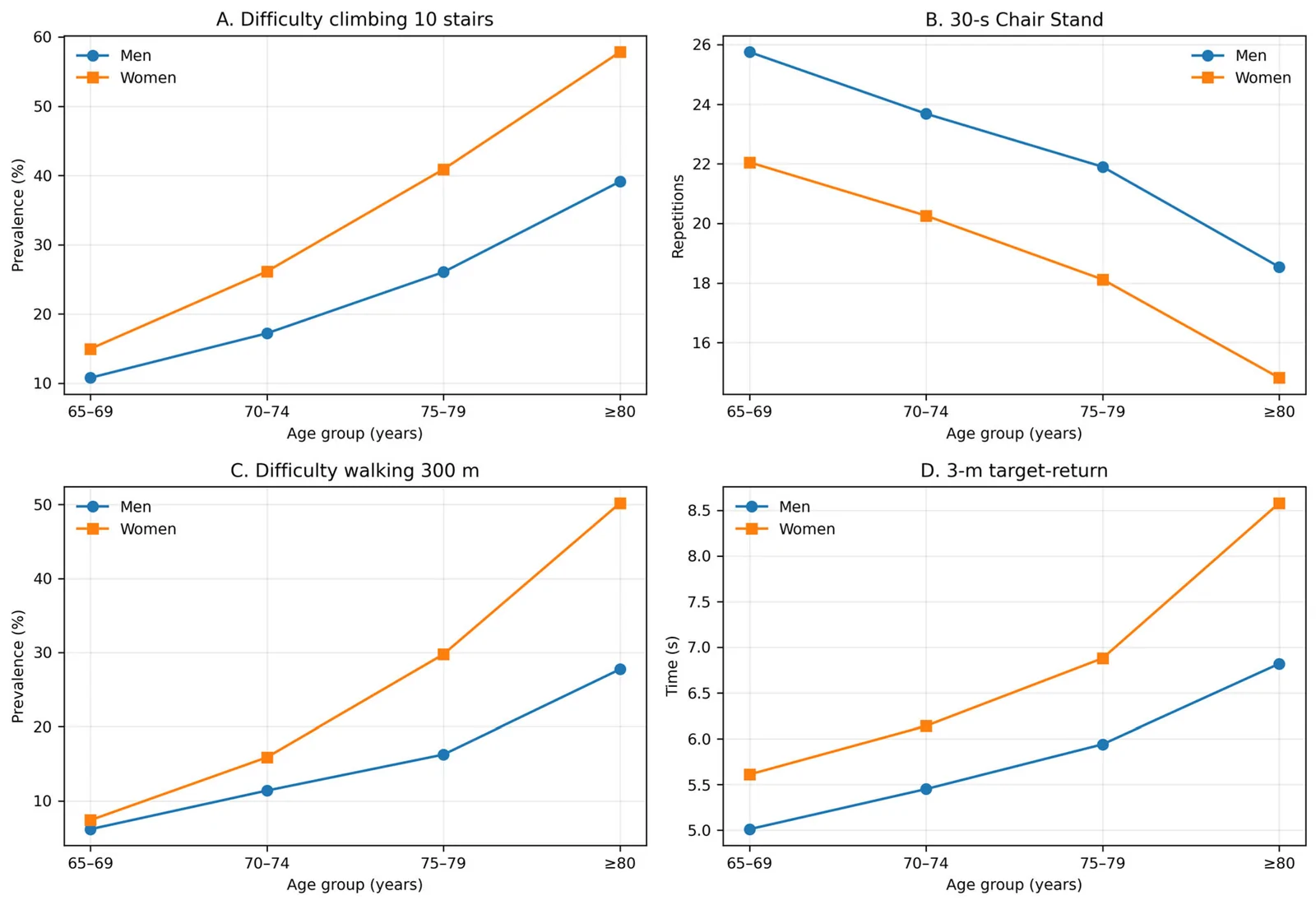
Separate age-by-sex descriptive profiles of self-reported limitations (NSOK) and performance (NFA). Higher limitation percentages indicate worse reported function; more Chair Stand repetitions indicate better performance, whereas longer 3 m target return times indicate worse performance. Panels use different samples and units, and no individual-level or between-dataset statistical agreement was tested.

For 300 m walking limitation, corresponding weighted prevalences were 6.17%, 11.39%, 16.25%, and 27.73% among men and 7.39%, 15.88%, 29.76%, and 50.18% among women. The age-by-sex interaction was statistically significant (Wald χ^2^ = 24.05, df = 3, *p* < 0.001), and the descriptive difference between the youngest and oldest age groups was greater among women (Table 2, Figure 1; Supplementary Table S2).

### 3.3. Age- and Sex-Specific Profiles of Exercise Behavior

Among men, exercise non-participation was 47.16% (95% CI 43.29–51.04%) at ages 65–69, 46.17% at 70–74, 40.88% (36.64–45.13%) at 75–79, and 50.58% at ≥80 years. The within-men omnibus age-group test was significant (Wald χ^2^ = 9.98, df = 3, *p* = 0.019), indicating variation among age strata rather than a monotonic age gradient. The exploratory 75–79 versus 65–69 comparison yielded an odds ratio of 0.775 (95% CI 0.623–0.964; unadjusted *p* = 0.022); it did not meet the 0.05 criterion after Holm adjustment across the three comparisons with the youngest group (adjusted *p* = 0.066; Supplementary Table S3). Among male exercisers, mean weekly exercise days were 4.40, 4.96, 4.98, and 4.90, respectively. Among women, non-participation was 42.47%, 42.76%, 45.67%, and 57.65% across the four age groups. The within-women age-group test was significant (Wald χ^2^ = 41.81, df = 3, *p* < 0.001), largely reflecting higher non-participation in the ≥80-year group; women aged 65–79 had more similar values. Mean days per week among female exercisers were 4.51, 4.70, 4.89, and 4.62. In an exploratory time-based description among exercisers, the weighted mean of reported exercise days multiplied by minutes per session was 255.8, 286.7, 267.5, and 257.7 min/week in men and 235.0, 226.8, 230.4, and 184.3 min/week in women. This proxy captures reported exercise time only; it is not an intensity-adjusted exercise dose. The original age-by-sex interaction for non-participation remained significant (Wald χ^2^ = 17.66, df = 3, *p* < 0.001).

### 3.4. Age- and Sex-Specific Profiles of Objective Functional Fitness

Objective functional fitness values were less favorable in older age groups in both sexes. Mean grip strength among men was 38.79 ± 5.78, 36.19 ± 5.69, 33.83 ± 5.83, and 30.33 ± 6.06 kg across the four age groups; among women, the corresponding values were 24.02 ± 4.19, 22.88 ± 4.25, 21.36 ± 4.23, and 19.15 ± 4.88 kg. The age-group-by-sex interaction for grip strength was statistically significant (*p* < 0.001), but its effect size was small (partial η^2^ = 0.0139).

Mean 30 s Chair Stand performance among men was 25.75 ± 5.40, 23.68 ± 5.83, 21.90 ± 6.01, and 18.54 ± 5.91 repetitions across the four age groups; the corresponding values among women were 22.05 ± 6.06, 20.26 ± 6.04, 18.12 ± 6.03, and 14.82 ± 5.36 repetitions. The age-group-by-sex interaction was not statistically significant (*p* = 0.282), and the estimated effect size was very small (partial η^2^ = 0.00015).

Mean 3 m target return times among men were 5.01 ± 0.95, 5.45 ± 1.12, 5.94 ± 1.36, and 6.82 ± 1.84 s across the four age groups; the corresponding values among women were 5.61 ± 1.06, 6.14 ± 1.33, 6.88 ± 1.69, and 8.58 ± 2.69 s. The age-group-by-sex interaction was statistically significant (*p* < 0.001), but the effect size was small (partial η^2^ = 0.0162; Table 3, Figure 1; Supplementary Table S7).

### 3.5. Contextual Directional Profiles Across Independent Data Sources

As context for the NSOK findings, the NFA showed less favorable Chair Stand and 3 m performance in older age categories, while NSOK stair-climbing and 300 m walking limitations were more prevalent in its older categories. For example, among women, the prevalence of 300 m walking limitation was 7.39% at ages 65–69 and 50.18% at ≥80 years, while NFA 3 m times in the same nominal categories were 5.61 and 8.58 s. These are expected age-related descriptive directions shared by many functionally relevant measures, not evidence that the selected pairings outperform other possible pairings or that the measures are interchangeable.

Exercise participation profiles were less consistently ordered across age strata than the NFA performance profiles. In NSOK men aged 65–79, non-participation was 47.16%, 46.17%, and 40.88% rather than showing a steady increase; over the corresponding NFA categories, grip strength, Chair Stand repetitions, and 3 m performance were less favorable in the older groups. Women aged 65–79 also had relatively similar non-participation prevalences, while the independent NFA records showed different descriptive fitness profiles. No inferential comparison of these heterogeneous cross-sample gradients was made. Because source populations, measurement domains, and selection processes differ, these observations do not demonstrate statistically established exercise–fitness discordance or an ecological association at the individual level.

### 3.6. Sensitivity Analysis

The NSOK outcomes were recalculated including 127 proxy respondents (*N* = 10,078). Maximum absolute changes from the self-respondent analysis were 1.40 percentage points for stair-climbing limitation, 1.68 for 300 m walking limitation, and 0.70 for exercise non-participation; age-by-sex directions were unchanged (Supplementary Table S1). Only 1.3% of respondents were proxies, so this check cannot rule out all proxy-related bias. The additional within-sex contrasts for exercise non-participation are reported in Supplementary Table S3. The male 75–79 versus 65–69 contrast was nominally significant before multiplicity adjustment but did not remain significant after Holm correction (adjusted *p* = 0.066). This distinction was retained in interpreting the descriptive exercise profiles.

## 4. Discussion

This analysis aligned age-by-sex profiles from two independent 2023 Korean datasets: self-reported task limitations and exercise behavior in the population-based NSOK, and objective performance among voluntary NFA assessment records. Reported task difficulty and objective performance were generally less favorable in older categories, as would be expected for age-sensitive functional indicators. The contribution is a description of how different information domains are distributed across the same nominal age-by-sex strata, not evidence of specific test-pair equivalence, individual agreement, or an exercise–fitness association.

The different source populations are the main obstacle to interpreting cross-dataset patterns. NSOK uses a population-based probability sample; NFA records were generated by persons who chose to undergo assessment at designated centers. The degree of selection may differ by age, sex, health, and motivation. For example, older persons who attend a fitness center could be functionally different from the wider population, but the direction or magnitude of this difference was not estimable using the provided extract. Consistent with this concern, the age-by-sex composition differed between weighted NSOK strata and NFA records (Supplementary Table S5). This demonstrates non-equivalence of the samples, not the direction of fitness-related selection. An external same-protocol, same-year benchmark was not part of the available analytic data. Historical KNHANES grip-strength estimates would require additional harmonization of test protocols, age bands, and survey design before numerical differences could be attributed to selection. Consequently, sampling effects cannot be separated from measurement-domain differences using the available data.

The lower extremity pairing illustrates the descriptive nature of the analysis. Stair-climbing difficulty and fewer Chair Stand repetitions both characterized older categories, but this resemblance is neither unique to the prespecified pair nor statistically tested against unrelated measures. Importantly, the stair-climbing age-by-sex interaction was not significant (*p* = 0.102), so sex differences in the observed absolute percentages should not be interpreted as proven differences in age-related patterns. The Chair Stand interaction was also not significant (*p* = 0.282), despite lower mean repetitions among women at each age stratum.

The mobility domain comparison has a larger construct mismatch. The 300 m question concerns sustained independent walking, whereas the 3 m target return test involves standing, short-distance movement, turning, and sitting. Its sequence resembles the Timed Up and Go test [16], but identical testing protocols cannot be assumed. Both measures had less favorable descriptive values in older strata, especially in women, yet such ordering alone does not validate one against the other. The interaction effect sizes for NFA grip and 3 m time were small (partial η^2^ = 0.0139 and 0.0162); their clinical importance remains undetermined.

Exercise behavior requires similarly restrained interpretation. Among men aged 65–79 years, non-participation did not follow a monotonic age gradient, while objective performance in independent NFA records was lower in older categories. The within-men omnibus age-group test was statistically significant, whereas the contrast between ages 75–79 and 65–69 did not remain significant after Holm adjustment (adjusted *p* = 0.066). Therefore, this is a descriptive difference in profile shape rather than demonstrated exercise–fitness discordance. Among exercisers, greater reported exercise frequency or minutes may reflect time availability after retirement, selective participation, or other factors, but retirement status as an explanation was not established by the analysis. The participation threshold—one session of at least 10 min a week—captures minimal engagement, not activity intensity, long-term exposure, or training quality. The derived weekly minutes measure likewise lacks intensity and was assessed only among exercisers.

Cross-sectional differences among age groups must not be described as changes occurring as individuals age. Cohort composition, health-related participation, survivor selection, and other unmeasured factors may contribute. The data also provide no basis for conclusions about exercise effectiveness: participants in separate data sources were not linked, and exercise dose was not experimentally controlled.

For community health assessment, the practical implication is conditional rather than prescriptive. A yes/no exercise item, difficulty climbing stairs, and performance tests address different constructs and should not be assumed to substitute for one another without individual-level validation. Research that measures all three within the same people is needed to establish whether combining them improves assessment, surveillance, or intervention decisions. The present findings alone cannot show that any of these indicators is routinely used as a proxy for functional capacity or that one method yields superior screening accuracy.

NFA data remain valuable as a large set of standardized program assessment records, even though they cannot be generalized to the entire older population. Their use here was deliberately limited to within-source descriptive profiles. Comparisons with national survey benchmarks may help characterize potential center selection bias in future research, but require comparable measurement definitions, matching age strata, and preferably similar calendar periods.

### Strengths and Limitations

The study has two practical strengths: independently reproducible descriptive results in two large 2023 sources and explicit separation of exercise behavior, task-specific reported difficulty, and objective performance. NSOK subgroup analyses incorporated its survey weight and PSU clustering, while NFA results retained record-level denominators. The supplementary analyses include sensitivity to proxy inclusion and within-sex exercise contrasts with multiplicity adjustment.

The central limitation is that the independent datasets represent different source populations. Treating differences between their aggregate age-by-sex profiles as individual-level relationships would be an ecological fallacy. Voluntary NFA participation may vary across strata, and selection bias could not be quantified from the provided variables. The NFA file lacks exact ages within its open-ended ≥80-year group, center or geographic identifiers, and identifiers needed to distinguish repeat visits; the 27,440 records cannot be interpreted as 27,440 unique people. Although NFA operates a structured certification program, site-specific adherence could not be verified. The 300 m limitation and three-meter target return test assess different tasks; the two-minute step test was absent from the provided extract, and the six-minute walk had only 0.67% valid records. NSOK exercise participation had a low threshold and did not capture intensity or cumulative exposure; the ancillary weekly minutes calculation relied on reported frequency and session duration among exercisers. NSOK public-use data also lacked an identifiable stratum variable. The original stratified design is documented [8], but the weight-plus-PSU approach could not reconstruct its full variance structure. Taken together, these constraints preclude claims of validated measurement similarity, association, predictive utility, or causality.

## 5. Conclusions

In two independent 2023 Korean datasets, task-specific reported limitations and objective functional performance were generally less favorable in older age categories. Exercise non-participation did not follow a similarly monotonic profile in every age-by-sex stratum, but the cross-source differences were descriptive and susceptible to selection and measurement differences. The highlighted within-men age contrast did not remain significant after multiplicity adjustment. These results provide context for the different information captured by exercise participation questions, task difficulty reports, and objective tests, not evidence that any measure can substitute for another. Studies collecting these domains with comparable procedures in the same individuals are needed to evaluate their associations, clinical value, and potential use together in older adult health assessment.

## Figures and Tables

**Table 1:** Analytic samples by sex and age group in the two data sources.

Sex	Age Group	NSOK Self-Respondents, *n* ^1^	NFA Assessment Records, *n* ^2^
Men	65–69	1176	6262
Men	70–74	1031	4082
Men	75–79	767	2328
Men	≥80	850	1409
Women	65–69	2067	5202
Women	70–74	1436	3843
Women	75–79	1166	2588
Women	≥80	1458	1726
Total	All age groups	9951	27,440

^1^ Only self-respondents were included in the primary NSOK analysis. ^2^ NFA values represent assessment records and should not be assumed to equal the number of unique individuals.

**Table 2:** Survey-weighted prevalences of limitations and exercise non-participation, and survey-weighted mean weekly exercise days among exercisers, by sex and age group in the 2023 NSOK.

Sex	Age Group	*n*	Difficulty Climbing 10 Stairs, % (95% CI)	Difficulty Walking 300 m, % (95% CI)	Exercise Non-Participation, % (95% CI)	Exercise Days/Week Among Exercisers (95% CI)
Men	65–69	1176	10.79 (8.45–13.13)	6.17 (4.61–7.74)	47.16 (43.29–51.04)	4.40 (4.24–4.56)
Men	70–74	1031	17.21 (14.70–19.71)	11.39 (9.33–13.44)	46.17 (42.49–49.85)	4.96 (4.80–5.12)
Men	75–79	767	26.05 (22.26–29.83)	16.25 (13.16–19.33)	40.88 (36.64–45.13)	4.98 (4.78–5.17)
Men	≥80	850	39.12 (34.36–43.88)	27.73 (23.96–31.49)	50.58 (45.70–55.46)	4.90 (4.69–5.11)
Women	65–69	2067	14.97 (12.82–17.11)	7.39 (6.06–8.72)	42.47 (39.56–45.38)	4.51 (4.39–4.63)
Women	70–74	1436	26.15 (23.21–29.10)	15.88 (13.53–18.22)	42.76 (39.29–46.23)	4.70 (4.56–4.84)
Women	75–79	1166	40.89 (37.05–44.73)	29.76 (26.20–33.32)	45.67 (41.86–49.48)	4.89 (4.73–5.05)
Women	≥80	1458	57.82 (53.72–61.93)	50.18 (46.19–54.17)	57.65 (53.60–61.71)	4.62 (4.41–4.83)

Note: Values are survey-weighted estimates using s_wgt1, except for n, which represents the unweighted number of self-respondents in each age-by-sex group. Functional limitations and exercise non-participation are reported as weighted prevalences (%), whereas exercise days per week are reported as weighted means among exercisers only. The denominators for weekly exercise days therefore differ from the total group *n*. The 95% confidence intervals (CIs) account for clustering at the survey-district level. Age-by-sex interactions were evaluated using 3-df omnibus Wald χ^2^ tests: stair-climbing limitation, χ^2^ = 6.20, *p* = 0.102; 300 m walking limitation, χ^2^ = 24.05, *p* < 0.001; and exercise non-participation, χ^2^ = 17.66, *p* < 0.001. Additional within-sex age-group comparisons, including Holm-adjusted *p* values, are presented in Supplementary Table S3.

**Table 3:** Objective functional fitness in the 2023 NFA data by sex and age group (mean ± SD).

Sex	Age Group	All Records, *n*	Grip *n*	Grip (kg)	Chair Stand *n*	30 s Chair Stand (Reps)	3 m *n*	3 m Target Return (s)
Men	65–69	6262	6262	38.79 ± 5.78	6069	25.75 ± 5.40	6071	5.01 ± 0.95
Men	70–74	4082	4082	36.19 ± 5.69	3993	23.68 ± 5.83	3993	5.45 ± 1.12
Men	75–79	2328	2328	33.83 ± 5.83	2290	21.90 ± 6.01	2289	5.94 ± 1.36
Men	≥80	1409	1409	30.33 ± 6.06	1385	18.54 ± 5.91	1381	6.82 ± 1.84
Women	65–69	5202	5201	24.02 ± 4.19	5022	22.05 ± 6.06	5030	5.61 ± 1.06
Women	70–74	3843	3843	22.88 ± 4.25	3755	20.26 ± 6.04	3756	6.14 ± 1.33
Women	75–79	2588	2588	21.36 ± 4.23	2545	18.12 ± 6.03	2543	6.88 ± 1.69
Women	≥80	1726	1725	19.15 ± 4.88	1703	14.82 ± 5.36	1698	8.58 ± 2.69

Note: All *n* values represent valid NFA assessment records for the indicated outcome, not necessarily unique persons. Missing values were not imputed. Means are reported as mean ± SD with units in column headings. Age-by-sex interaction (HC3): grip strength, *p* < 0.001, partial η^2^ = 0.0139; 30 s Chair Stand, *p* = 0.282, partial η^2^ = 0.00015; 3 m target return, *p* < 0.001, partial η^2^ = 0.0162.

## Data Availability

The 2023 National Survey of Older Koreans public-use microdata were accessed through the KIHASA Health and Welfare Data Portal (https://data.kihasa.re.kr/) on 25 August 2026, subject to the provider’s data-access application procedures. The 2023 National Fitness Award microdata used in this study were obtained through Statistics Korea’s Microdata Integrated Service (https://mdis.kostat.go.kr/) and accessed on 1 September 2026, subject to the provider’s registration and application procedures. No new participant-level data were generated for this secondary analysis.

## Supplementary Materials

The following supporting information can be downloaded at: https://www.mdpi.com/article/10.3390/healthcare14213699/s1, Table S1: Sensitivity analysis including proxy respondents in the NSOK; Table S2: Age-group-by-sex interaction estimates from weighted logistic regression models in the NSOK; Table S3: Within-sex NSOK age-group contrasts for exercise non-participation; Table S4: Available data and missingness in the 2023 datasets; Table S5: Age-by-sex composition in independent NSOK and NFA sources; Table S6: Age-group, sex, and age-by-sex interaction tests in the NSOK; Table S7: Age-group, sex, and age-by-sex interaction tests for objective functional fitness in the NFA.

## Abbreviations

The following abbreviations are used in this manuscript:

NFA	National Fitness Award
NSOK	National Survey of Older Koreans
