# Supplementary material for: Age- and Sex-Specific Profiles of Self-Reported Functional Limitations, Exercise Behavior, and Objective Functional Fitness in Korean Older Adults: Evidence from Two Independent 2023 Data Sources
Source: Healthcare (Basel). 2026 Nov 1;14(21):3699. doi: 10.3390/healthcare14213699 (PMC13762835; doi:10.3390/healthcare14213699)
Supplement: Supplementary file 1 [file healthcare-14-03699-s001.zip › healthcare-4623770-supplementary.pdf]

Supplementary Materials — Healthcare Manuscript healthcare-4623770

Age- and Sex-Specific Profiles of Self-Reported Functional Limitations, Exercise Behavior, and Objective Functional Fitness in Korean Older Adults: Evidence from Two Independent 2023 Data Sources

Table S1. Sensitivity analysis including proxy respondents in the NSOK.

Sex	Age group	Outcome	Primary self-respondent analysis (%)	All 10,078 respondents (%)	Difference (percentage points)
Men	65–69	Stair limitation	10.79	10.88	+0.09
Men	65–69	300-m limitation	6.17	6.32	+0.15
Men	65–69	Exercise non-participation	47.16	47.17	+0.00
Men	70–74	Stair limitation	17.21	17.73	+0.53
Men	70–74	300-m limitation	11.39	11.77	+0.38
Men	70–74	Exercise non-participation	46.17	46.12	−0.05
Men	75–79	Stair limitation	26.05	26.24	+0.19
Men	75–79	300-m limitation	16.25	16.64	+0.39
Men	75–79	Exercise non-participation	40.88	41.32	+0.44
Men	≥80	Stair limitation	39.12	40.52	+1.40
Men	≥80	300-m limitation	27.73	29.40	+1.68
Men	≥80	Exercise non-participation	50.58	51.03	+0.46
Women	65–69	Stair limitation	14.97	15.04	+0.07
Women	65–69	300-m limitation	7.39	7.47	+0.08
Women	65–69	Exercise non-participation	42.47	42.52	+0.05
Women	70–74	Stair limitation	26.15	26.20	+0.04
Women	70–74	300-m limitation	15.88	15.86	−0.02
Women	70–74	Exercise non-participation	42.76	42.68	−0.08
Women	75–79	Stair limitation	40.89	41.20	+0.31

Sex	Age group	Outcome	Primary self-respondent analysis (%)	All 10,078 respondents (%)	Difference (percentage points)
Women	75–79	300-m limitation	29.76	30.16	+0.40
Women	75–79	Exercise non-participation	45.67	45.92	+0.25
Women	≥80	Stair limitation	57.82	59.09	+1.27
Women	≥80	300-m limitation	50.18	51.52	+1.33
Women	≥80	Exercise non-participation	57.65	58.35	+0.70

Note: Difference = analysis including all respondents minus the primary self-respondent analysis. Maximum absolute differences were 1.40 percentage points for stair-climbing limitation, 1.68 percentage points for 300-m walking limitation, and 0.70 percentage points for exercise non-participation. Differences were calculated before rounding; subtracting the displayed two-decimal percentages may differ by 0.01 percentage points.

Table S2. Age-group-by-sex interaction estimates from weighted logistic regression models in the NSOK.

Outcome	Age-group contrast	β	SE	Interaction OR	95% CI	p
Stair-climbing limitation	70–74 vs 65–69	0.158	0.153	1.171	0.868–1.581	0.301
Stair-climbing limitation	75–79 vs 65–69	0.300	0.165	1.350	0.977–1.864	0.069
Stair-climbing limitation	≥80 vs 65–69	0.383	0.167	1.466	1.058–2.032	0.022
300-m walking limitation	70–74 vs 65–69	0.191	0.195	1.211	0.826–1.775	0.327
300-m walking limitation	75–79 vs 65–69	0.588	0.205	1.800	1.205–2.690	0.004
300-m walking limitation	≥80 vs 65–69	0.772	0.188	2.164	1.496–3.132	<0.001
Exercise non-participation	70–74 vs 65–69	0.052	0.119	1.053	0.834–1.329	0.663
Exercise non-participation	75–79 vs 65–69	0.385	0.132	1.470	1.135–1.903	0.004
Exercise non-participation	≥80 vs 65–69	0.475	0.141	1.609	1.220–2.122	<0.001

Note: The reference category was men aged 65–69 years. Each interaction OR indicates how the female–male difference in the stated age group differed from the female–male difference at age 65–69 years. Omnibus age-group-by-sex interaction: stair-climbing limitation, χ^2 = 6.20 (df = 3), p = 0.102; 300-m walking limitation, χ^2 = 24.05 (df = 3), p < 0.001; exercise non-participation, χ^2 = 17.66 (df = 3), p < 0.001. Models were weighted binomial logistic regressions using s_wgt1 with cluster-robust sandwich covariance by surveyNoR.

Table S3. Within-sex NSOK age-group contrasts for exercise non-participation.

Sex	Age contrast	OR	95% CI	Raw p	Holm p
**Men**	70–74 vs 65–69	0.961	0.792–1.165	0.685	0.685
**Men**	75–79 vs 65–69	0.775	0.623–0.964	0.022	0.066
**Men**	≥80 vs 65–69	1.146	0.903–1.455	0.261	0.522
**Women**	70–74 vs 65–69	1.012	0.862–1.187	0.886	0.886
**Women**	75–79 vs 65–69	1.139	0.957–1.355	0.144	0.287
**Women**	≥80 vs 65–69	1.844	1.511–2.251	<0.001	<0.001

Reference: 65–69 years within the same recorded sex. Weighted logistic regressions used s_wgt1 and a PSU-clustered sandwich covariance by surveyNoR. Holm adjustment was performed separately over the three pairwise age comparisons within each sex. Confidence intervals are unadjusted 95% intervals. The male within-sex age-group omnibus test was Wald $\chi^2(3) = 9.98$, $p = 0.019$; the corresponding female test was $\chi^2(3) = 41.81$, $p < 0.001$. The interaction was Wald $\chi^2(3) = 17.66$, $p < 0.001$. These analyses did not reconstruct strata unavailable in the public-use file.

Table S4. Available data and missingness in the 2023 datasets.

Dataset/outcome	Available records	Missing / not available	Interpretation
**NSOK stair item (C4)**	9,951	0	Valid 1/2 response codes among self-respondents
**NSOK walking item (C5)**	9,951	0	Valid 1/2 response codes among self-respondents
**NSOK exercise participation (B12)**	9,951	0	Valid yes/no response codes among self-respondents
**NSOK exercise days (B12-1)**	5,339 exercisers	4,612 skips	Structural skip for non-exercisers (code 9)
**NSOK exercise minutes (B12-2)**	5,339 exercisers	4,612 skips	Structural skip for non-exercisers (code 999)
**NFA grip strength**	27,438	2 (0.01%)	Available records
**NFA 30-s Chair Stand**	26,762	678 (2.47%)	Available records
**NFA 3-m target-return**	26,761	679 (2.47%)	Available records
**NFA 6-min walk**	183	27,257 (99.33%)	Excluded because coverage was insufficient
**NFA 2-min step**	Variable absent	Not determinable	Not present in provided extract/specification

The NFA file consists of 27,440 assessment records rather than identifiable unique persons. The public-use NFA extract contains categorical age codes only (including an open-ended ≥80 category), and contains no person, visit, center or geographical identifier. NSOK self-respondents aged ≥80 had an unweighted mean age of 83.81 years (median 83, n = 2,308); comparable values cannot be estimated from the NFA extract.

Among NSOK exercisers, weighted mean reported weekly exercise minutes (reported days × minutes/session) for men were 255.8, 286.7, 267.5, and 257.7 across age groups 65–69, 70–74, 75–79, and ≥80 years, respectively; the corresponding values among women were 235.0, 226.8, 230.4, and 184.3. These descriptors are conditional on exercise participation and do not measure intensity or total physical activity.

Table S5. Age-by-sex composition in independent NSOK and NFA sources.

Recorded sex	Age group	NSOK weighted within-sex %	NFA within-sex record %	NFA records
**Men**	65–69	38.22	44.47	6262
**Men**	70–74	25.19	28.99	4082
**Men**	75–79	17.42	16.53	2328
**Men**	≥80	19.17	10.01	1409
**Women**	65–69	32.13	38.94	5202
**Women**	70–74	22.57	28.77	3843
**Women**	75–79	17.33	19.37	2588
**Women**	≥80	27.98	12.92	1726

The NSOK weighted male proportion was 44.04%; the NFA male share was 51.32% of records. NFA values are nonprobability records and may include repeated measurements from the same person. These composition differences do not identify the direction or magnitude of fitness-related selection bias.

Table S6. Age-group, sex, and age-by-sex interaction tests in the NSOK.

NSOK outcome	Men age Wald χ^2	Men p	Women age Wald χ^2	Women p	Sex @65–69 χ^2	Sex p	Interaction p
**Stair-climbing limitation**	128.01	<0.001	380.71	<0.001	9.34	0.002	0.102
**300-m walking limitation**	127.20	<0.001	567.42	<0.001	1.45	0.229	<0.001
**Exercise non-participation**	9.98	0.019	41.81	<0.001	5.76	0.016	<0.001

NSOK: Age tests within each sex are 3-df joint Wald tests; sex is the female–male reference-group contrast at ages 65–69 (1 df), not a population-average sex main effect; the age×sex interaction is 3 df. Each test used the weighted GLM and a PSU-clustered sandwich covariance; the full strata structure was unavailable.

Table S7. Age-group, sex, and age-by-sex interaction tests for objective functional fitness in the NFA.

NFA outcome	Age Wald $\chi^2(3)$	Sex Wald $\chi^2(1)$	Age×sex Wald $\chi^2(3)$	Interaction p
**Grip strength**	4607.85	32322.32	370.56	<0.001
**30-s Chair Stand**	4377.60	2085.10	3.82	0.282
**3-m target-return**	5025.35	1630.78	222.56	<0.001

Note: NFA effects are Wald tests from HC3-robust OLS models with sum-to-zero contrasts; age and recorded sex main effects for the three outcomes all had $p < 0.001$. These record-level models cannot address repeat visits because participant identifiers were unavailable.
